# Intrabiliary Rupture of a Hepatic Hydatid Cyst Causing Mechanical Biliary Obstruction: A Case Report of Endoscopic Management With a Self-Expandable Metal Stent

**DOI:** 10.7759/cureus.116483

**Published:** 2026-09-18

**Authors:** Razan A Awad, Khulood K Al-Dahash, Snaha Mariyam Abraham

**Affiliations:** 1 Department of Emergency Medicine, Tawam Hospital, Al Ain, ARE

**Keywords:** albendazole therapy, case report, complex hydatid cyst, cystic echinococcosis (ce), cystobiliary communication, endoscopic retrograde cholangiopancreatography (ercp), hepatic hydatid cyst, intrabiliary rupture

## Abstract

A 27-year-old Afghan male with no significant medical history presented with a one-month history of epigastric abdominal pain and nausea. Four weeks prior to presentation, he had been diagnosed with a hepatic hydatid cyst in Afghanistan and was prescribed albendazole but was nonadherent to treatment and follow-up. At presentation, he was afebrile and had no clinical jaundice. Contrast-enhanced computed tomography of the abdomen demonstrated an 8.0 cm hydatid cyst in the left hepatic lobe with direct communication with the left hepatic duct and associated dilatation of the intrahepatic biliary ducts and common bile duct, which measured 15 mm. Hyperattenuating material within the distal common bile duct, consistent with impacted hydatid material, resulted in mechanical biliary obstruction, with findings consistent with intrabiliary rupture of the hepatic hydatid cyst. The patient was treated with albendazole and subsequently underwent endoscopic retrograde cholangiopancreatography (ERCP), during which membranous hydatid material was observed emerging from the major papilla. Endoscopic sphincterotomy and balloon sweeping were performed to remove the obstructing material, followed by placement of a self-expandable metal stent for biliary drainage. The patient demonstrated clinical improvement, with resolution of common bile duct dilatation on follow-up ultrasonography. Subsequent hepatic resection of the involved lobes was performed five weeks after ERCP. This case highlights that intrabiliary rupture of a hepatic hydatid cyst may cause significant mechanical biliary obstruction despite the absence of overt clinical jaundice and demonstrates the therapeutic role of ERCP in achieving biliary decompression.

## Introduction

*Echinococcosis*, commonly known as hydatid disease, is a zoonotic parasitic infection caused predominantly by *Echinococcus granulosus*, which is responsible for cystic echinococcosis, and *Echinococcus multilocularis*, which causes alveolar echinococcosis [1]. Humans are accidental intermediate hosts who acquire the infection through ingestion of *Echinococcus *eggs from contaminated food or water or through contact with infected animals [1]. The liver is the most frequently affected organ, accounting for approximately 70% of cases, followed by the lungs, which are involved in approximately 20-30% of cases [2].

Hepatic hydatid cysts may remain clinically silent for years and often become symptomatic when complicated by rupture, infection, or involvement of adjacent structures [1,2]. Rupture into the biliary tree is an important complication of hepatic hydatid disease and may result in cystobiliary communication, an abnormal connection between the hydatid cyst and the biliary tree that allows cyst contents to enter the bile ducts. Frank intrabiliary rupture refers to the direct rupture of the cyst into the bile ducts, with extrusion of hydatid membranes or other cyst contents into the biliary system. This may cause mechanical biliary obstruction, obstructive jaundice, cholangitis, or pancreatitis [3,4].

The diagnosis of hepatic hydatid disease is primarily based on radiological imaging, supported by serological investigations when appropriate [2]. Imaging is particularly important when biliary involvement is suspected, as it may demonstrate biliary dilatation, cystobiliary communication, or intraductal hydatid material [3]. Endoscopic retrograde cholangiopancreatography (ERCP) has an important therapeutic role in patients with frank intrabiliary rupture and biliary obstruction, allowing biliary decompression and removal of obstructing hydatid material [3,5].

We report a case of hepatic hydatid disease complicated by cystobiliary communication and intrabiliary rupture, resulting in mechanical common bile duct (CBD) obstruction due to impacted hydatid material despite the absence of overt clinical jaundice or cholangitis. The patient was managed with endoscopic sphincterotomy, extraction of the obstructing hydatid material, and placement of a self-expandable metal stent (SEMS). The objective of this case report is to highlight the importance of recognizing biliary involvement in hepatic hydatid disease and to describe the therapeutic role of ERCP and self-expandable metal stent placement in relieving mechanical biliary obstruction.

## Case presentation

A 27-year-old Afghan male with a known hepatic hydatid cyst presented to the hospital with a one-month history of severe epigastric abdominal pain associated with nausea. Notably, despite the eventual identification of biliary obstruction, he had no clinical features of obstructive jaundice or cholangitis. He had experienced similar symptoms in his home country, where he was diagnosed with a hepatic hydatid cyst and was prescribed albendazole but was nonadherent to treatment or follow-up.

At presentation to the emergency department, he reported severe epigastric pain, rated 9/10 in intensity, which was non-radiating and associated with nausea without vomiting. He denied fever, jaundice, pruritus, dark urine, pale stools, or weight loss. Review of systems was otherwise unremarkable. He had no significant surgical history or known drug allergies, and no family history of similar complaints. He reported recent travel and contact with farm animals, including cattle, during the preceding three months in his home country.

On physical examination, he appeared well and was hemodynamically stable, with a temperature of 36.0°C, blood pressure of 117/78 mmHg, heart rate of 98 beats/min, and respiratory rate of 18 breaths/min. There was no pallor, jaundice, or peripheral edema. The abdomen was soft and non-distended, with mild, non-radiating epigastric tenderness. There was no hepatosplenomegaly, palpable abdominal mass, ascites, or evidence of peritonism. 

Initial laboratory investigations demonstrated mixed hepatocellular and cholestatic abnormalities with associated hyperbilirubinemia, in addition to mild hypokalemia, as shown in Table 1.

**Table 1:** Laboratory investigations at presentation. ALP, alkaline phosphatase; ALT, alanine aminotransferase; ANC, absolute neutrophil count; AST, aspartate aminotransferase; Cl, chloride; Cr, creatinine; Hb, hemoglobin; Hct, hematocrit; K, potassium; MCV, mean corpuscular volume; PLT, platelet count; TBil, total bilirubin; WBC, white blood cell count. Initial laboratory investigations demonstrated significant elevations in transaminases, with an ALT of 376 IU/L and AST of 272 IU/L, accompanied by an elevated ALP of 234 IU/L and total bilirubin of 47.7 µmol/L. These findings were consistent with hepatocellular and cholestatic liver abnormalities with associated hyperbilirubinemia. Mild hypokalemia was also present, with a serum potassium level of 3.4 mmol/L. The remaining hematological and biochemical parameters were within the laboratory reference ranges.

Parameter	Result	Reference range
WBC	8.3 × 10⁹/L	4.0-11.0 × 10⁹/L
ANC	6.14 × 10⁹/L	2.0-7.5 × 10⁹/L
Hb	140 g/L	120-160 g/L
Hct	0.40 L/L	0.36-0.46 L/L
MCV	80.1 fL	80-100 fL
PLT	232 × 10⁹/L	150-400 × 10⁹/L
ALP	234 IU/L	40-130 IU/L
ALT	376 IU/L	7-56 IU/L
AST	272 IU/L	10-40 IU/L
TBil	47.7 µmol/L	5-21 µmol/L
K	3.4 mmol/L	3.5-5.1 mmol/L
Cl	105 mmol/L	98-107 mmol/L
Cr	49.5 µmol/L	45-90 µmol/L

An initial abdominal ultrasound (US) demonstrated a well-defined complex cystic lesion in the left hepatic lobe (Figure 1A), approximately 6.1 × 6.4 × 5.9 cm, with an estimated volume of 121 mL. The lesion demonstrated cystic changes and posterior acoustic enhancement, with surrounding vascularity. The portal vein remained patent with preserved flow (Figure 1B). Marked biliary dilatation was also noted, with the common bile duct (CBD) measuring up to 16 mm and associated intrahepatic biliary duct dilatation (Figure 1C).

**Figure 1:**
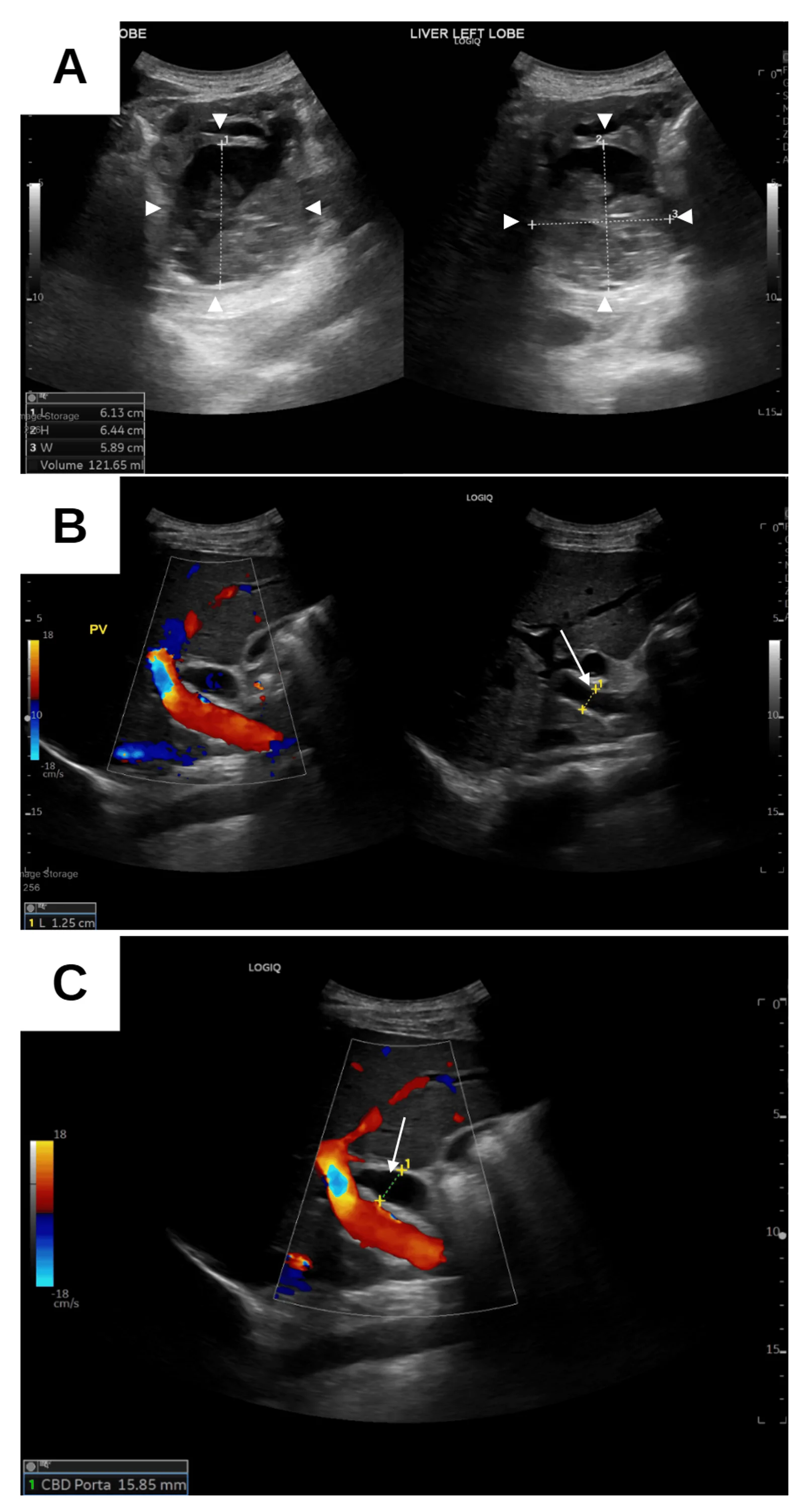
Ultrasonographic findings of hepatic hydatid disease with biliary obstruction. (A) A well-defined complex cystic lesion in the left hepatic lobe (arrowheads), measuring 6.1 × 6.4 × 5.9 cm (volume: 121 mL), with internal cystic changes and posterior acoustic enhancement. (B) Color Doppler ultrasound showing a patent portal vein (PV) with preserved blood flow, measuring 1.25 cm in diameter (arrow). (C) Color Doppler ultrasound showing marked dilatation of the common bile duct (CBD) at the porta hepatis, measuring approximately 16 mm in diameter (arrow).

Based on the clinical, laboratory, and initial imaging findings, a complicated hepatic hydatid cyst with biliary obstruction was suspected. The infectious diseases (ID) team recommended albendazole 400 mg twice daily and further evaluation with *Echinococcus *serology and chest computed tomography (CT). Contrast-enhanced CT of the abdomen demonstrated an 8.0 cm cystic lesion in the left hepatic lobe communicating directly with a dilated left hepatic duct. The CBD was markedly dilated, measuring 15 mm, with findings consistent with cystobiliary communication and intrabiliary rupture (Figure 2A). Hyperattenuating material in the distal CBD was consistent with impacted hydatid contents, causing mechanical biliary obstruction. The left portal and hepatic venous branches were not visualized, likely secondary to compression or encasement. The cyst abutted the diaphragm near the inferior pericardium and left cardiophrenic angle (Figure 2B); however, there was no evidence of transdiaphragmatic extension or pulmonary hydatid disease on chest CT. *Echinococcus *serology was subsequently positive at a titer of 1:1280.

**Figure 2:**
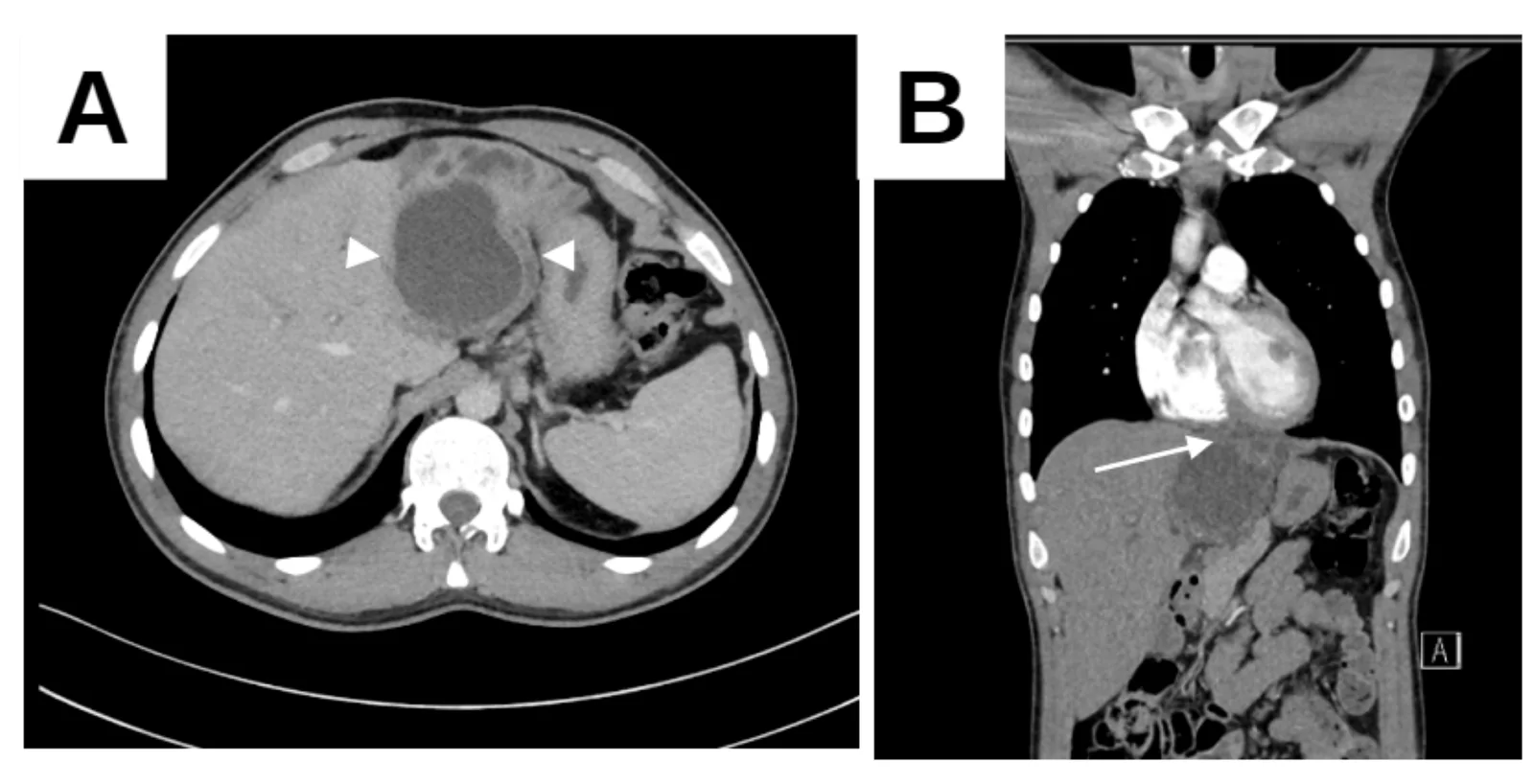
Contrast-enhanced computed tomography (CT) demonstrating hepatic hydatid disease and its thoracoabdominal extent. (A) Axial CT demonstrates a large complex hydatid cyst in the left hepatic lobe (arrowheads). (B) Coronal CT shows the superior aspect of the cyst abutting the diaphragm and the inferior pericardial region (arrow), without transdiaphragmatic extension.

A multidisciplinary team (MDT) comprising infectious diseases, general surgery, internal medicine, gastroenterology, and radiology recommended endoscopic intervention to relieve the biliary obstruction. ERCP was subsequently performed for diagnostic assessment and therapeutic management. During the procedure, membranous, whitish material was observed emerging from the major papilla, consistent with hydatid material (Figure 3A). Endoscopic sphincterotomy was performed, followed by balloon sweeping of the CBD, resulting in removal of the obstructing hydatid material (Figure 3B). A SEMS was subsequently placed to maintain biliary patency and facilitate ongoing biliary drainage (Figure 3C).

**Figure 3:**
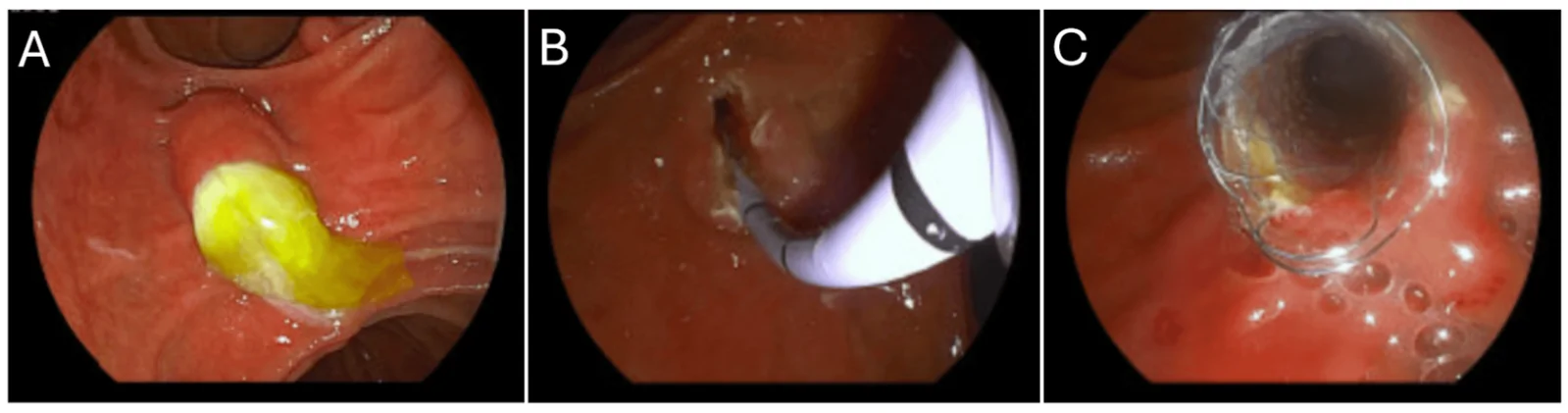
Endoscopic findings during ERCP for intrabiliary rupture of a hepatic hydatid cyst. (A) Whitish membranous hydatid material protruding from the major papilla. (B) Endoscopic extraction of intraductal hydatid material following sphincterotomy and common bile duct balloon sweeping. (C) Placement of a self-expandable metallic biliary stent to maintain biliary drainage. ERCP: endoscopic retrograde cholangiopancreatography.

The patient remained clinically stable following ERCP, with marked improvement in abdominal pain, resolution of nausea, and tolerance of oral intake. Post-ERCP laboratory investigations showed improvement in AST and ALT, while bilirubin and ALP increased. Serum lipase decreased and remained within the reference range, with preserved renal function (Table 2).

**Table 2:** Laboratory parameters before and after ERCP. ERCP, endoscopic retrograde cholangiopancreatography; Na, sodium; K, potassium; CO₂, carbon dioxide; ALP, alkaline phosphatase; AST, aspartate aminotransferase; ALT, alanine aminotransferase; eGFR, estimated glomerular filtration rate.

Laboratory parameter	Pre-ERCP	Post-ERCP	Normal reference range
Na (mmol/L)	136	136	135-145
K (mmol/L)	3.4	3.8	3.5-5.0
CO₂ (mmol/L)	23	20	22-29
Lipase (IU/L)	41	16	13-60
Albumin (g/L)	31	31	35-50
Total bilirubin (µmol/L)	47.7	62.6	5-21
Direct bilirubin (µmol/L)	32.4	45.7	0-5
ALP (IU/L)	234	297	40-129
AST (IU/L)	272	110	10-40
ALT (IU/L)	376	237	7–56
Creatinine (µmol/L)	49.5	55.2	62-106
eGFR (mL/min/1.73 m²)	142	136	≥90

Follow-up hepatobiliary US demonstrated a reduction in cyst volume to approximately 83 mL and marked improvement of the previously observed CBD dilatation (Figure 4A). The difference in cyst measurements between ultrasonography and computed tomography may be attributable to differences in imaging modality, measurement technique, and the plane of measurement. The biliary stent remained in situ, and the CBD measured approximately 7 mm, compared with 16 mm before intervention (Figures 4B, 4C).

**Figure 4:**
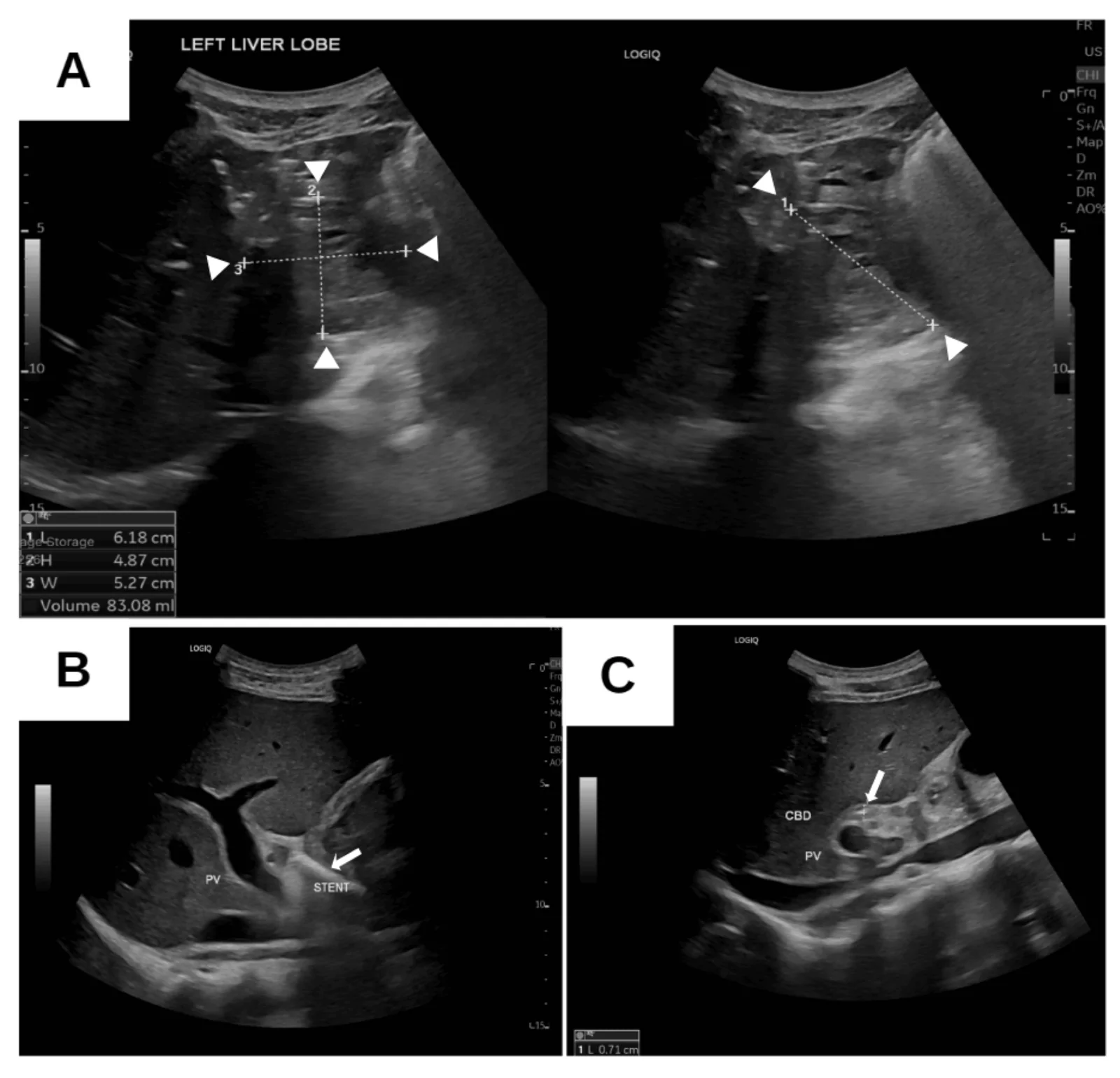
Follow-up ultrasonographic findings after ERCP and biliary stent placement. (A) Residual complex cystic lesion (arrowheads) in the left hepatic lobe measuring approximately 6.2 × 4.9 × 5.3 cm (volume, 83 mL), demonstrating interval reduction in size compared with the initial examination. (B) Visualization of the indwelling biliary stent (arrow) adjacent to the portal vein (PV). (C) Common bile duct (arrow) measuring approximately 7 mm, demonstrating resolution of the previously observed biliary dilatation. ERCP: endoscopic retrograde cholangiopancreatography; CBD: common bile duct.

Given his continued clinical improvement, resolution of the CBD dilatation on imaging, and no indication for acute surgical intervention, the patient was deemed clinically fit for discharge the following day. He was discharged on albendazole 400 mg twice daily with instructions for outpatient follow-up. At outpatient follow-up with the ID team on day 19 after ERCP, he remained asymptomatic with no new complaints and was advised to continue albendazole therapy. Four weeks after ERCP, his case was reviewed at an MDT meeting. Given the residual hepatic cyst despite medical and endoscopic management, hepatic resection was recommended. The patient was informed of the treatment plan and provided consent for surgery.

One week later, the patient underwent open resection of hepatic segments II and III. Intraoperative US was performed, and the cyst was identified within the left lateral hepatic section. During parenchymal transection, the left hepatic duct was encountered and found to be partially open at the site corresponding to the known cystobiliary communication. The duct was divided and oversewn. The left portal venous branch and left hepatic vein were also divided to complete the resection. Two small bile leaks from the resection surface were identified and oversewn, and a subhepatic drain was placed. Intraoperative US demonstrated no residual biliary dilatation. Estimated blood loss was 50 mL. The resected specimen comprised hepatic segments II and III containing the hydatid cyst.

Histopathological examination showed dilated bile ducts lined by cuboidal epithelium, surrounded by fibrous and fibrovascular tissue, with marked chronic active inflammation and extensive fibrosis. Numerous eosinophils were present within the inflammatory infiltrate. No hydatid cyst membrane, granuloma, or malignancy was identified in the examined sections. The postoperative course was clinically stable, with no documented complications in the available records. Albendazole therapy was to be continued for six more weeks. Long-term follow-up data were unavailable, limiting assessment of the patient’s long-term clinical outcome and recurrence.

## Discussion

Laboratory findings in cystic echinococcosis (CE) are generally normal or nonspecific, although eosinophilia may be present [1, 6]. The diagnosis of CE is based on clinical findings, imaging, and serology, with US serving as the primary imaging modality and CT providing complementary anatomical detail, particularly for assessing complications [2, 7]. Serology can be used to support the diagnosis; however, a negative result does not exclude CE [8].

Cystobiliary communication is a recognized complication of hepatic CE and may result in biliary obstruction, obstructive jaundice, cholangitis, or pancreatitis. Cystobiliary communication is classified as simple or frank intrabiliary rupture based on the extent of communication between the cyst and the biliary tree [5]. Minor communications may remain clinically silent, whereas major communication with frank intrabiliary rupture allows hydatid membranes and cyst contents to enter the bile ducts, potentially causing partial or complete biliary obstruction [3,9]. Frank intrabiliary rupture has been reported in approximately 3-17% of patients with hepatic hydatid cysts [9]. A recent systematic review of 102 studies involving 1,241 patients with cholangiohydatidosis identified obstructive jaundice and acute suppurative cholangitis as the most common clinical presentations, occurring in 51.4% and 42.6% of patients, respectively [10]. In contrast, our patient presented with marked intrahepatic and extrahepatic biliary dilatation and biochemical evidence of biliary obstruction but remained afebrile and without clinically apparent jaundice or cholangitis.

ERCP confirmed frank intrabiliary rupture by demonstrating whitish membranous hydatid material emerging through the major papilla. Similar endoscopic findings have been described in frank intrabiliary rupture, including hydatid membranes within the bile ducts or protruding through the ampulla of Vater [9]. ERCP is particularly valuable in this setting because it provides both diagnostic confirmation and therapeutic biliary decompression through sphincterotomy and endoscopic extraction of obstructing hydatid material [9,11].

Endoscopic management of biliary complications of hepatic hydatid disease has been described in several clinical series. Akaydin et al. reported 70 ERCP procedures in 54 patients, including endoscopic sphincterotomy, hydatid membrane extraction, biliary drainage, and stent placement, with improvement in biochemical abnormalities following treatment [11]. Borahma et al. reported 16 patients with hydatid cyst rupture into the biliary tree who underwent endoscopic sphincterotomy followed by balloon or basket extraction and, when required, biliary drainage. Biliary fistula closure was achieved in 80% of patients, with a median healing time of six weeks [12]. More recently, Gümüşoğlu et al. reported 38 patients with major intrabiliary rupture managed with endoscopic sphincterotomy and evacuation of hydatid contents, followed by either nasobiliary drainage or biliary stenting [13]. In our patient, ERCP with endoscopic sphincterotomy, balloon extraction of obstructing hydatid material, and biliary stent placement was similarly effective in relieving the biliary obstruction, with subsequent resolution of CBD dilatation and clinical improvement.

The use of a SEMS in the present case is a notable feature. Published series of major intrabiliary ruptures have predominantly described endoscopic sphincterotomy with extraction of hydatid material, followed by nasobiliary drainage or plastic biliary stenting [12,13]. Reports describing SEMS use in hydatid-related biliary disease appear to be limited. García-Cano et al. described SEMS placement for a large intrahepatic biliary fistula following hydatid cyst surgery, with cessation of external biliary drainage within 24 hours [14]. However, that case involved a postoperative biliary fistula rather than spontaneous frank intrabiliary rupture with common bile duct obstruction. In our patient, endoscopic sphincterotomy and balloon extraction of hydatid material, followed by SEMS placement, achieved effective biliary decompression, with the common bile duct decreasing from 16 mm before intervention to 7 mm on follow-up US.

The favorable early response following ERCP should nevertheless be interpreted cautiously, as biliary decompression does not necessarily constitute definitive treatment of the underlying hepatic disease. In the series by Gümüşoğlu et al., 16 of 23 patients who underwent biliary stenting required subsequent ERCP because of persistent jaundice or cholangitis, while six required percutaneous drainage because of infection of the residual hydatid cavity [13]. Despite these additional interventions, all patients ultimately achieved complete recovery during a mean follow-up of 23 months [13]. In the present case, ERCP successfully relieved the acute biliary obstruction; however, a residual hepatic cyst remained. Definitive resection of segments II and III was therefore performed approximately five weeks later. Importantly, surgery directly demonstrated the cystobiliary communication with the left hepatic duct, corroborating the preoperative imaging and endoscopic findings.

This case has several limitations. The duration of hydatid disease before presentation could not be established, and the patient had not completed the initial course of albendazole because of nonadherence. Most importantly, long-term follow-up was unavailable, limiting assessment of recurrence, subsequent biliary complications, and the long-term outcomes of endoscopic and surgical treatment. Nevertheless, this case highlights the successful short-term endoscopic management of frank intrabiliary rupture with mechanical biliary obstruction. The favorable clinical and radiological response following hydatid material extraction and SEMS placement suggests that SEMS may have a role in maintaining biliary drainage in selected cases; however, further evidence is needed to determine its optimal use and long-term efficacy in hydatid-related biliary complications.

## Conclusions

This case highlights that frank intrabiliary rupture of a hepatic hydatid cyst can cause significant mechanical biliary obstruction even in the absence of overt jaundice or cholangitis. Multimodal imaging helped define the cystobiliary communication and biliary obstruction, while ERCP enabled direct visualization and removal of obstructing hydatid material, followed by SEMS placement to maintain biliary drainage. The patient demonstrated prompt clinical improvement and resolution of CBD dilatation following endoscopic intervention. However, ERCP addresses the biliary complication rather than the underlying hepatic disease and continued clinical and radiological follow-up is important to assess cyst evolution, closure of the cystobiliary communication, stent removal, and possible recurrence. 
